# Neglected food-borne trematodiases: echinostomiasis and gastrodiscoidiasis

**DOI:** 10.1017/S0031182022000385

**Published:** 2022-09

**Authors:** Rafael Toledo, María Álvarez-Izquierdo, J. Guillermo Esteban, Carla Muñoz-Antoli

**Affiliations:** Área de Parasitología, Departamento de Farmacia y Tecnología Farmacéutica y Parasitología, Facultad de Farmacia, Universitat de València, Avda, Vicent Andrés Estellés s/n, 46100 Burjassot, Valencia, Spain

**Keywords:** Echinostomatidae, echinostomes, food-borne trematode infections, Gastrodiscidae, *Gastrodiscoides hominis*, intestinal trematodes

## Abstract

In the present paper, we review two of the most neglected intestinal food-borne trematodiases: echinostomiasis, caused by members of the family Echinostomatidae, and gastrodiscoidiasis produced by the amphistome *Gastrodiscoides hominis*. Both parasitic infections are important intestinal food-borne diseases. Humans become infected after ingestion of raw or insufficiently cooked molluscs, fish, crustaceans, amphibians or aquatic vegetables. Thus, eating habits are essential to determine the distribution of these parasitic diseases and, traditionally, they have been considered as minor diseases confined to low-income areas, mainly in Asia. However, this scenario is changing and the population at risk are currently expanding in relation to factors such as new eating habits in developed countries, growing international markets, improved transportation systems and demographic changes. These aspects determine the necessity of a better understanding of these parasitic diseases. Herein, we review the main features of human echinostomiasis and gastrodiscoidiasis in relation to their biology, epidemiology, immunology, clinical aspects, diagnosis and treatment.

## Graphical abstract

**Figure:**
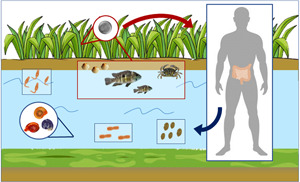

## Introduction

Digenea is a class of Trematoda that comprises a large group of organisms with significant medical and veterinary interest. Over 100 species of digenetic trematodes have been reported infecting humans, many of them transmitted through food. Food-borne trematodiases constitute one of the most neglected tropical diseases group and includes liver flukes, lung flukes and intestinal flukes. Commonly, these parasitic infections have been ignored both in terms of research funding and presence in the public media. More than 40 million people are currently infected and about 10% of the world's population live at risk of infection (Keiser and Utzinger, 2009; Sripa *et al*., 2010; Toledo *et al*., 2019). In the past, these infections were limited for the most part in populations living in low-income countries, particularly in southeast Asia, and were associated with poverty. However, the geographical limits and the population at risk are currently expanding and changing in relation to factors such as growing international markets, improved transportation systems and demographic changes.

The present review focuses on two intestinal food-borne trematodiases of importance: echinostomiasis, caused by members of the family Echinostomatidae, and gastrodiscoidiasis produced by the amphistome *Gastrodiscoides hominis*. These parasitic diseases are associated with practices of eating raw or insufficiently cooked molluscs, fish, crustaceans, amphibians, aquatic plants, promiscuous defecation and the use of human excrements collected from latrines for fertilization of ponds. They are aggravated by socioeconomic factors including poverty, malnutrition, a growing free-food market, a lack of supervised food inspection, poor or insufficient sanitation, other helminthiases and declining economic conditions. In this context, further studies are urgently needed to clarify the current epidemiology of these helminth infections and to identify new and specific targets for both effective diagnosis and treatments (Graczyk and Fried, 1998; Chai, 2009; Toledo and Esteban, 2016; Toledo *et al*., 2019).

Herein, we will summarize the main features of the biology, clinical, epidemiology and current diagnostic tools of these food-borne trematode infections and the corresponding diseases they cause.

## Echinostomiasis

**Fig. 1.**
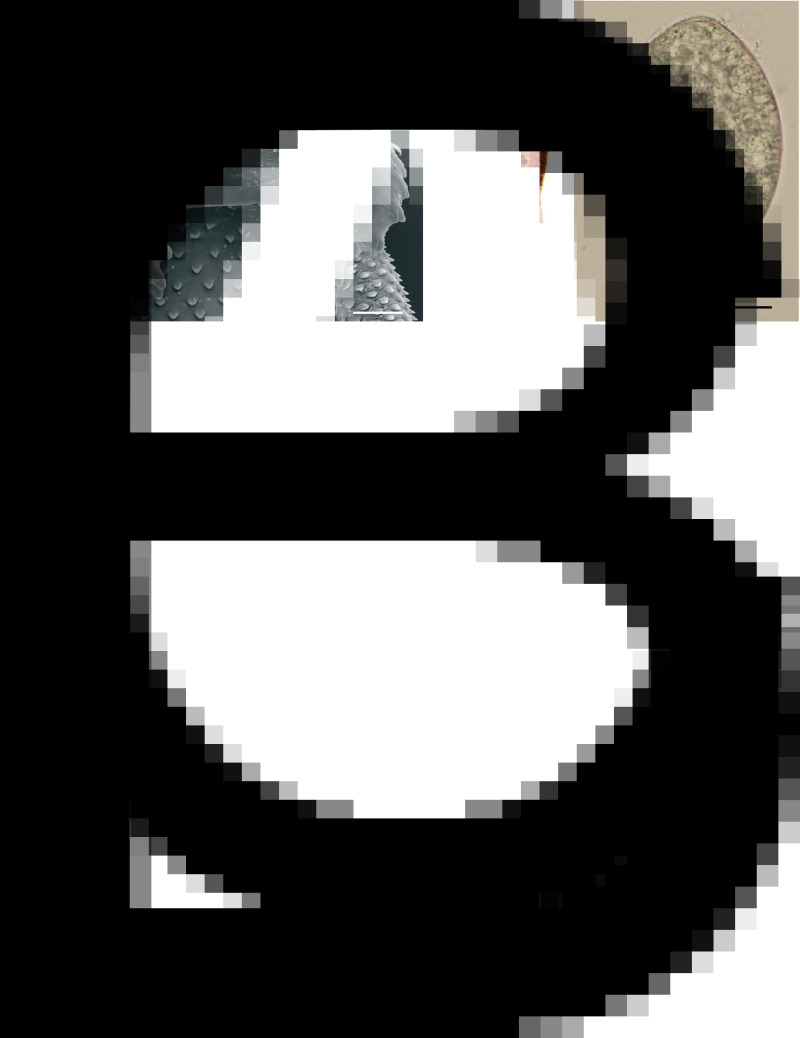
*Echinostoma* sp.: (A) scanning electron micrographs of the cephalic collar of spines (scale bar: 100 *μ*m); (B) adult worm (scale bar: 1 mm) and (C) unembryonated egg (scale bar: 10 *μ*m).

Echinostomiasis is the parasitic disease caused by echinostomes. Under this term are included the trematodes belonging to the family Echinostomatidae. This family constitutes a heterogeneous group of hermaphroditic digeneans comprising 50 genera and more than 350 species (Yamaguti, 1971; Chai and Jung, 2020). Adult echinostomes commonly inhabit the intestine of a wide range of birds and mammals, including humans. Eventually, some species of echinostomes have been also recorded in reptiles and fishes (Toledo and Esteban, 2016; Toledo *et al*., 2019). Echinostomes are characterized by the presence of a head collar of spines arranged in one or two circles around the oral sucker. The number and disposition of the spines constitute an important taxonomic feature (Fig. 1). However, systematics of echinostomes is very complex and a subject of continuous reviews and phylogenetic analyses (Chai *et al*., 2020*a*; Izrailskaia *et al*., 2021; Pham *et al*., 2022).

Although echinostomes are distributed worldwide, human echinostomiasis mainly occurs at endemic foci in southeast Asia and Far East due to the eating habits of those areas. However, this scenario may be changing due to several factors such as changes in the eating habits in Western countries, internationalization of food markets, migratory flows and the progressive improvement of transportation systems (Graczyk and Fried, 1998; Chai, 2009; Toledo and Esteban, 2016; Toledo *et al*., 2019).

### Morphology and life cycle

Adult worms of the family Echinostomatidae are characterized by the presence of a head collar with collar spines around the oral sucker (Fig. 1A). The number and arrangement of collar spines are important features for taxonomic purposes. Considerable variation exists in size of echinostomes depending upon species, fixation procedures, definitive host and crowding effect and size ranges about 2–10 × 1–2 mm^2^. The spines of the cephalic collar may be arranged in one or two circles and the number of spines is constant within the species. The tegument contains scale-like spines on both dorsal and ventral surfaces, though the number and size of the spines are reduced in the posterior half of the body. The oral and ventral suckers are close to each other. The two testes, usually in tandem, are posterior to the ovary. The uterus is intercaecal and normally pre-ovarian. The vitellarium is follicular, in two lateral fields, usually in the hindbody but may extend into the forebody (Fig. 1B).

**Fig. 2.**
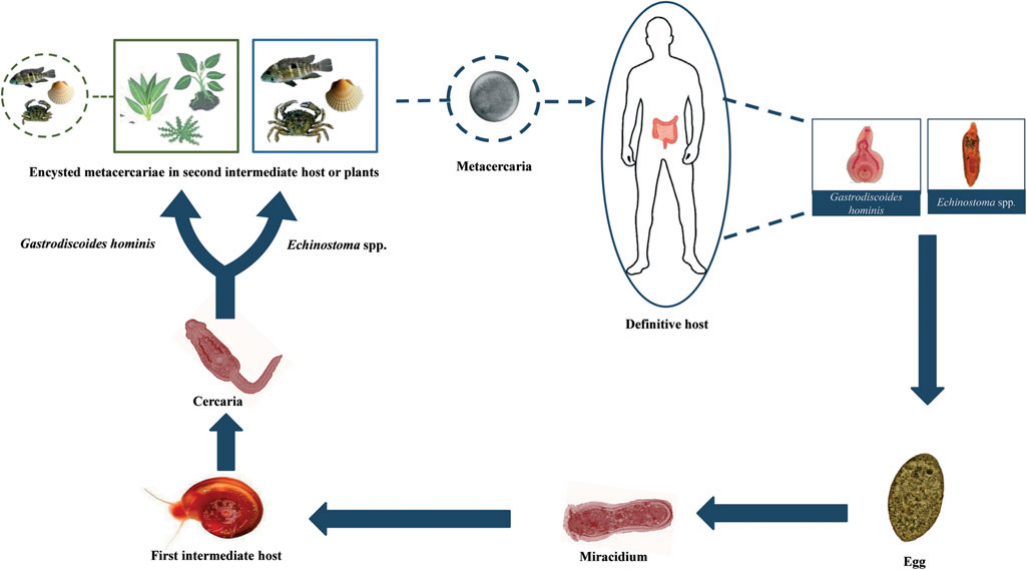
Generalized life cycle of echinostomes and *Gastrodiscoides hominis*: adult worms inhabit the small intestine of several vertebrate hosts, including humans; eggs are voided with host feces and miracidia hatch in freshwater and actively infect the snail first intermediate host; cercariae are released by the first intermediate host and swim to locate the second intermediate host (snails, amphibians, bivalves and fishes) which they penetrate in the case of echinostomes and on aquatic plants or, alternatively, aquatic animals in the case of *G. hominis*; cercariae become metacercariae after encystation; metacercariae are ingested by the definitive host and excyst to become adults.

Members of the Echinostomatidae follow a three-host life cycle (Fig. 2). The first intermediate hosts are aquatic snails in which subsequently a single generation of sporocysts, two generations of rediae and, finally, cercariae develop within the second generation of rediae. After emergence of the cercariae in an aquatic environment, they infect the second intermediate host, which may be several species of snails, clams, frogs and even fishes in which cercariae encyst to form the metacercariae. The definitive host becomes infected after eating of the second intermediate host harbouring the encysted metacercariae (Huffman and Fried, 1990; Fried *et al*., 2004; Toledo *et al*., 2009).

Strikingly, Miao *et al*. (2021) recently detected an ectopic echinostomiasis. An adult worm of *Echinostoma* sp. was detected in the urinary bladder of a 67-year-old man in Zhejiang (China) by cystoscopy. The patient complained of haematuria and dysuria but he did not show intestinal symptoms. The authors suggested that the metacercariae could penetrate through the intestinal wall into the urinary bladder, where the parasite attained maturity.

### Epidemiology

Human echinostome infections are a common feature since ancient times (Seo *et al*., 2020). Currently, human echinostomiasis is endemic in southeastern and northeastern Asia. However, the number and identity of the species involved in human infections are difficult to ascertain in relation to: (1) problematical taxonomy of the family that complicates the specific description due to continuous synonymies and misidentifications; (2) a lack of systematic surveys and (3) most of the reports are based on occasional records with uncertain, incomplete or lacking specific identifications, even today. For example, Li *et al*. (2019) detected eggs of an echinostome in the feces of a 42-year-old man from the northern area of China that was simply diagnosed as a member of Echinostomatidae. Similarly, Sah *et al*. (2018) reported the first case of human echinostomiasis in Nepal in a 62-year-old male resident in Gorkha district caused by an unidentified member of the genus *Echinostoma*. Consumption of insufficiently cooked snails and fishes appeared to be the cause of infection. Khanna *et al*. (2019) recorded a single case attributed to an *Echinostoma* species in a 3-year-old male child with severe anaemia in Karnataka (India). In fact, most recent reviews differ in the number of species causing this parasitic disease. Toledo and Esteban (2016) compiled a total of 23 species belonging to 9 different genera of Echinostomatidae. Toledo *et al*. (2019) listed a total of 20 species but, more recently, Chai and Jung (2020) considered 15 major species (having more than 6 reports each) and other 8 species (with fewer than 5 reports). Although these difficulties exist, we have listed a total of 23 species of echinostomes that have been recorded in humans, together with their main features. Table 1 compiles these species, together with the geographical locations of human infections.

**Table 1.** Species of Echinostomatidae involved in human infections and geographical location of cases (only those reports with specific description have been considered)

*Acanthoparyphium tyosenense*	Korea
*Artyfechinostomum malayanum*^a^	Cambodia, India, Indonesia, Lao PDR, Malaysia, Philippines, Singapore, Thailand
*Artyfechinostomum oraoni*	India
*Artyfechinostomum sufrartyfex*	India
*Echinochasmus caninus*^b^	Thailand
*Echinochasmus fujianensis*	China
*Echinochasmus japonicus*	China, Korea, Japan^c^, Lao PDR
*Echinochasmus jiufoensis*	China
*Echinochasmus liliputanus*	China
*Echinochasmus perfoliatus*	China, Japan, Korea
*Echinoparyphium recurvatum*	Egypt, Indonesia, Korea, Taiwan
*Echinostoma aegyptica*	China
*Echinostoma angustitestis*	China
*Echinostoma cinetorchis*	China, Japan, Korea, Taiwan
*Echinostoma ilocanum*	Cambodia, China, India, Indonesia, Malaysia, Philippines, Thailand
*Echinostoma lindoense*^d^	Indonesia, Lao PDR
*Echinostoma macrorchis*	Japan, Korea, Lao PDR
*Echinostoma mekongi*	Cambodia
*Echinostoma revolutum*	Cambodia, China, Egypt, Indonesia, Lao PDR, Russia, Europe
*Himasthla muehlensi*	USA^e^
*Hypoderaeum conoideum*	Thailand
*Isthmiophora hortensis*^f^	China, Japan, Korea
*Isthmiophora melis*	China, Romania, Taiwan

a Syn. *Echinostoma malayanum*.

b Syn. *Episthmium caninum*.

c Experimental infection.

d Syn *Echinostoma echinatum*.

e Imported infection.

f Syn. *Echinostoma hortense*.

Although echinostomes are spread worldwide, the source of infection strongly determines the geographical distribution of human echinostomiasis. Humans become infected after eating raw or undercooked freshwater or brackish water molluscs, fishes, snakes, crustaceans and amphibians (tadpoles or frogs) harbouring the infective metacercariae (Carney, 1991; Toledo *et al*., 2012, 2019; Toledo and Esteban, 2016; Chai and Jung, 2020). Thus, echinostomiasis mainly occurs in areas where traditional cultural practices encourage ingestion of those types of foods such as Asia, mainly in southeastern and northeastern Asia. Commonly, echinostomiasis is highly focal and most of the cases have been reported in China, India, Indonesia, Korea, Malaysia, the Philippines, Russia, Taiwan and Thailand (Chai, 2009; Toledo and Esteban, 2016). Occasional cases have also been reported in other countries (see Table 1).

The current incidence of human echinostomiasis is not known. Most of the available information is based on sporadic reports with scarce information and, in many cases, lacking specific identification as mentioned above. Moreover, microscopists may misinterpret echinostome eggs, particularly considering that eggs of different echinostome species markedly resemble making difficult specific diagnosis (Esteban *et al*., 2019; Chai *et al*., 2020*b*). The only estimate available is the one made by WHO (2004) for *Echinostoma hortense* (currently synonym of *Isthmiophora hortensis*) (50 000 people infected), *Echinochasmus japonicus* (about 5000 cases) and *Echinostoma cinetorchis* and *Acanthoparyphium tyosenense* (with about 1000 cases each). This evidences the need for systematic epidemiological surveys, especially in areas where consumption of raw or undercooked intermediate hosts of echinostomes is a common habit.

As mentioned above, most of the cases of human echinostomiasis have been reported in Asia and commonly appear as endemic foci in areas where the presence of intermediate host and the natural definitive hosts (mainly waterfowls) coexist with the human practice of eating undercooked intermediate hosts (Toledo and Esteban, 2016). Exceptionally, drinking of contaminated water harbouring metacercariae may be the source of infection (Xiao *et al*., 1995). There are no data on the seasonality of the transmission, but it cannot be discarded as those that occurred with other trematodes (Kopolrat *et al*., 2020).

The highest number of echinostome species has been reported in China. A total of 13 species has been recorded in this country, especially in provinces of southeast China such as Fujian, Guangdong, Liaoning, Anhui, Yunnan and Hubei (Toledo *et al*., 2019). *Echinochasmus liliputanus* was the first species discovered in humans from China. A prevalence of 13.4% was reported in Anhui in 1991 (Yu and Mott, 1994) and, therefore, more than 2500 human infections have been recorded in this province (Xiao *et al*., 2005). In Fujian province, *Echinochasmus fujianensis* appears to be the most common species attaining a prevalence of 1.6–7.8% among residents of 5 areas, children from 3 to 15-year-old being the most affected population segment with about two-thirds of all cases (Yu and Mott, 1994). In some counties of Guangdong and Fujian, *E. japonicus* is a common human infection with prevalences of 4.9%, whereas *Echinochasmus perfoliatus* has been usually reported attaining prevalences of 1.8% (Yu and Mott, 1994). Human infections with other echinostome species in China are more anecdotical. *Echinostoma revolutum* has been found in Yunnan and Guangdong provinces and 6 patients who had eaten raw loach were found to be infected with *I. hortensis* in Liaoning province (Chen *et al*., 1993). Moreover, a single case of infection with *Echinochasmus jiufoensis* was detected during the autopsy of a 6-month-old girl in Guangzhou (Liang and Ke, 1988).

A total of five species has been reported in humans from Indonesia, mainly in the major islands (Sumatra, Java and Sulawesi). Infections were related to the local habit of eating raw bivalves (*Corbicula* spp.) from lakes. An average prevalence of 43%, but attaining 96%, of *Echinostoma lindoense* was reported from 1937 to 1956 in residents in the lake Lindu Valley (Sulawesi) (Carney *et al*., 1980). Moreover, in the area of Borneo the finding of echinostome eggs in stool specimens of residents was common (Cross and Basaca-Sevilla, 1986). The popularity of Korean and Japanese restaurants in Indonesia led to an increase in the number of cases (Kusharyono and Sukartinah, 1991).

In Lao PDR, Eom *et al*. (2014) estimated a prevalence of 0.7%. A total of 6 species of echinostomes has been found infecting humans, with special incidence in Riparian villages along the Mekong river. In Khammouane province, a prevalence of 1.1% of echinostome eggs belonging to *E. revolutum*, *Artyfechinostomum malayanum*, *E. japonicus* and *Echinoparyphium recurvatum* was detected in the feces of residents (Chai *et al*., 2012). Moreover, Sayasone *et al*. (2009) detected three human cases of *E. japonicus*. *Echinochasmus caninus* has been recently recorded for the first time in Lao PDR infecting humans. Cases of *E. japonicus* were associated with eating raw or insufficiently cooked freshwater fish in a traditional food locally known as ‘lap-pa’, ‘Koi-pa’ or ‘som-pa’. A total of 11 cases in Riparian people was detected in Khammouane (Chai *et al*., 2019). All the patients were co-infected other intestinal helminths. In Savannakhet province, Sayasone *et al*. (2009) detected three cases. Recently, several cases have been additionally reported in this province. Chai *et al*. (2018) recorded the first human case of *Echinostoma ilocanum* in Lao PDR and, moreover, *Echinostoma aegyptica* was found in five Riparian people for the first time in this country (Chai *et al*., 2020*b*). In southern Lao PDR, a prevalence of 6% was estimated by Sayasone *et al*. (2011).

Several species of echinostomes have been discovered infecting humans in Cambodia (Table 1). A prevalence of 1% of *E. ilocanum* was estimated in the Oddar Meanchey province (Sohn *et al*., 2011*a*) and of 7.5–22.4% of *E. revolutum* in schoolchildren from the Pursat province causing gastrointestinal symptoms (Chai *et al*., 2009, 2018; Sohn *et al*., 2011*b*). The source of infection appeared to be the eating of undercooked snails or clams sold on the way back home from school (Sohn *et al*., 2011*b*). Recently, a new species (*Echinostoma mekongi*) has been described on the basis of adult flukes collected from Riparian people residing along the Mekong river. Parasites were detected in six Riparian people from the localities of Kratie and Takeo (Cho *et al*., 2020).

In Taiwan, three species of echinostomes have been recorded with a prevalence ranging from 0.11 to 0.65% in some areas (Lu, 1982; Carney, 1991). A total of four species has been found in Thailand, seven species in Korea, four different species in India and two in the Philippines (Table 1). However, most of these cases were occasional.

Human echinostomiasis is very uncommon outside Asia. Moreover, most of these cases were imported, occurring mainly in Asian refugees or tourists to Africa (DeGirolami and Kimber, 1983; Poland *et al*., 1985; Chai, 2007; Chunge and Chunge, 2017).

### Immunology

Little is known about the immunology of human echinostomiasis. Most of the current knowledge on immunology of echinostome infections is based on experimental studies in laboratory rodents. Echinostomes induce strong antibody responses both at systemic and mucosal levels (Graczyk and Fried, 1994; Cho *et al*., 2007; Sotillo *et al*., 2007, 2014). However, this response markedly depends on the host species. At the serum level, energic immunoglobulin M (IgM), total IgG, IgG1 and IgG3 have been observed in mice infected with *Echinostoma caproni*, but, in contrast, only weak responses were observed in rats (Sotillo *et al*., 2007). In *I. hortensis*-infected mice an elevation of IgG1, IgE and IgA was detected (Cho *et al*., 2007). At the mucosal level, intense responses of IgM, IgA, IgG1 and IgG2a were reported in *E. caproni*-infected mice and only elevated levels of IgG2a were observed in rats (Sotillo *et al*., 2007). A total of four proteins (enolase, aldolase, actin and the 70 kDa heat-shock protein) was found to be the most immunogenic antigens (Sotillo *et al*., 2007). Moreover, the glycosylation of parasite antigens appears to be essential for antibody responses, suggesting that T-independent responses are of great importance (Sotillo *et al*., 2014). Strikingly, it has been shown that echinostomes are able to trap and degrade antibodies bound to their surface by secreted cathepsin L-like peptidases (Cortés *et al*., 2017*a*, 2019).

Resistance to echinostome infections has been traditionally associated with the development of Th2 responses, mainly mediated by interleukin 4 (IL-4) and/or IL-13 (Brunet *et al*., 2000; Toledo and Fried, 2005; Sotillo *et al*., 2011; Cortés *et al*., 2017*b*; Toledo *et al*., 2019). However, recent studies suggest that resistance depends on the ability of the host to maintain the mucosal homoeostasis in a process mediated by IL-25 (Muñoz-Antoli *et al*., 2016; Álvarez-Izquierdo *et al*., 2020*a*, 2020*b*). Resistance is associated with hyperplasia of local tuft cells and production of IL-25 which operates by facilitating the maintenance of the epithelial homoeostasis that could be ultimately responsible for parasite rejection (Muñoz-Antoli *et al*., 2016; Cortés *et al*., 2017*b*; Álvarez-Izquierdo *et al*., 2020*a*, 2020*b*).

*Echinostoma* spp. also induces changes at the cellular level and in the expression of certain glycoconjugates in the intestinal mucosa (Toledo *et al*., 2006*a*). Mastocytosis, eosinophilic infiltration and increase in the goblet cells and mast cell populations have been observed (Fujino *et al*., 1993, 1996*a*, 1996*b*; Fujino and Fried, 1993, 1996; Kim *et al*., 2000; Park *et al*., 2005; Toledo *et al*., 2006*b*; Muñoz-Antoli *et al*., 2007; Ryang *et al*., 2007). Moreover, alterations of the terminal sugar of the mucins produced by goblet cells may regulate the worm expulsion (Fujino and Fried, 1993, 1996; Park *et al*., 2005).

### Clinical manifestations

In heavy infections, echinostomes may cause more severe symptoms than other intestinal trematodes. Commonly, major clinical symptoms of human echinostomiasis may include unspecific symptoms such as diarrhoea, loss of body weight, epigastric and abdominal pain or easy fatigue. However, additional symptoms may include local eosinophilia, anaemia, oedema and anorexia (Graczyk and Fried, 1998; Toledo *et al*., 2006*a*, 2019; Toledo and Esteban, 2016). Recently, severe anaemia has been associated with echinostomiasis in a 3-year-old male child concomitantly infected with *Echinostoma* sp., *Trichuris trichiura* and *Ascaris lumbricoides* in India (Khanna *et al*., 2019). In *I. hortensis*-infected patients, nausea and vomiting, headache, acid belching and urinary incontinence were observed (Chang *et al*., 2005). Mucosal damage may include signs of chronic gastritis, infiltration of inflammatory cells, mucosal erosion and bleeding and lesions similar to adenocarcinoma (Chang *et al*., 2005).

In ectopic locations such as the urinary bladder, infection was characterized by haematuria with 305 red blood cells per *μ*L (normal 0–13), 297 white blood cells per *μ*L (normal 0–9) and a positive result of 2+ for occult blood in the urine. Moreover, haematuria was accompanied by urgency and dysuria (Miao *et al*., 2021).

### Diagnosis and treatment

Laboratory diagnosis of echinostomiasis is based on the finding of eggs in feces. The eggs are yellow-brown and thin-shelled with an operculum, and a slight thickening of the shell at the abopercular end. They are unembryonated when passed in feces. The size of human-infecting echinostome eggs ranges 66–145 *μ*m × 43–90 *μ*m (Esteban *et al*., 2019). However, differentiation of echinostome species, and even from other trematodes, on the basis of the egg morphology is difficult and recovery and identification of adult worms if possible is recommended. The similarity among the eggs of other trematodes must be taken into account to avoid confusion, especially with those that are carcinogenic such as *Opisthorchis viverrini* (Crellen *et al*., 2021). Occasionally, human echinostomiasis has been revealed by gastroduodenal endoscopy performed in relation to severe epigastric symptoms and ulcerative lesions in the stomach and duodenum (Lee and Hong, 2002; Cho *et al*., 2003; Park and Kim, 2006; Jung *et al*., 2014).

Praziquantel is the drug of choice for intestinal fluke infections although it is not included in the US product labelling for these infections. A single dose of 25 mg kg^−1^ of praziquantel is recommended for treatment of intestinal fluke infections. Echinostome infections can be treated successfully with slightly lower – single, oral 10–20 mg kg^−1^ praziquantel (Chai *et al*., 2009). Praziquantel is 90% absorbed after ingestion and then rapidly metabolized in the liver and the hydroxylated and conjugated products are excreted mainly in the urine within 24 h. Side-effects are minimal and include abdominal pain, nausea, headache and dizziness (WHO, 1995).

## Gastrodiscoidiasis

Gastrodiscoidiasis is a plant-borne parasitic disease caused by the intestinal amphistome *G. hominis*. Adult worms are pyramidal in shape and measure 8–14 × 5.5–7.5 mm^2^ (Mas-Coma *et al*., 2005, 2006). Morphologically, they are characterized by a dorsoventrally flattened body with an anterior part short and cylindrical and the posterior portion larger and discoidal. The most characteristic features of *G. hominis* include a subterminal pharynx, tandem, lobed testes, a post-testicular ovary, an ascending uterus and a ventral genital pore (Mas-Coma *et al*., 2005, 2006).

### Life cycle

**Fig. 3.**
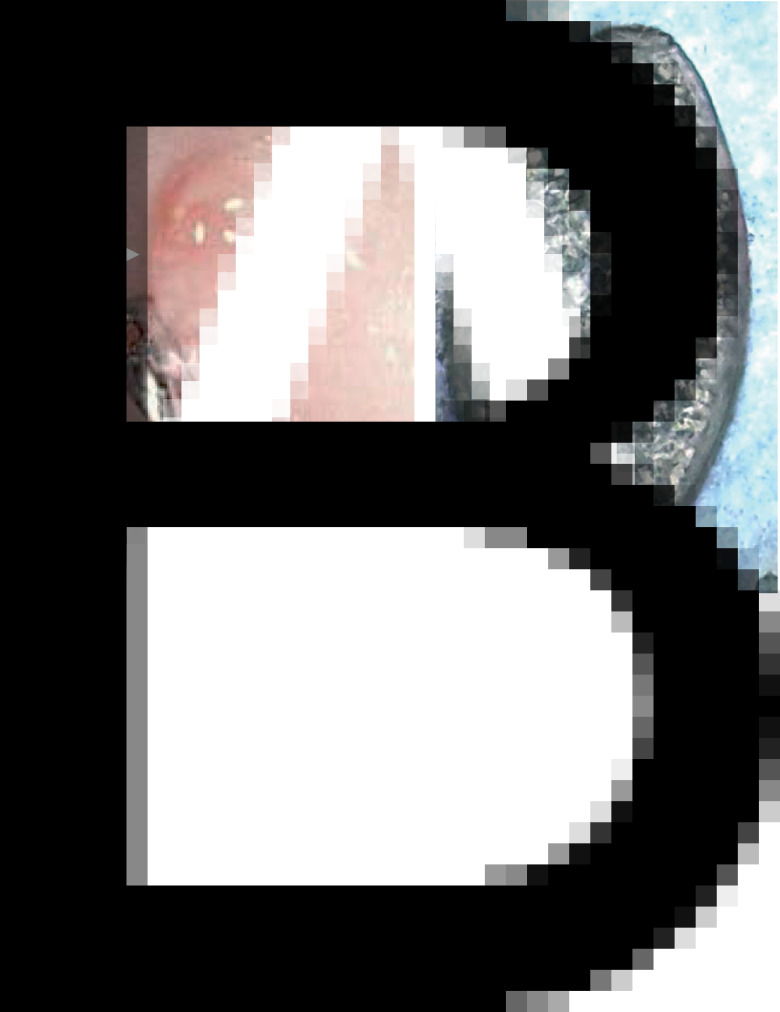
*Gastrodiscoides hominis*: (A) image of an upper endoscopy showing a juvenile living adult worm fixed in the mucosa of a human patient (arrow) and (B) egg isolated from the stools of a human patient (scale bar: 10 *μ*m). Photomicrographs: courtesy of Dr Ranjit Sah.

Life cycle of *G. hominis* is not well known (Fig. 2). Adult flukes inhabit the caecum and colon of several mammals, where it remains attached to the mucosa (Fig. 3A). Pigs appear to be the normal definitive host (Dutt and Srivastava, 1972), but it also has been reported in monkeys, mice, rats, wild boars, muskrats and humans (Easwaran *et al*., 2003; Mas-Coma *et al*., 2005, 2006). Unembryonated eggs (Fig. 3B) are laid in freshwater environments and a miracidium hatches and swims to infect the first intermediate host. Only the aquatic snail *Helicorbis coenosus* is known to act as the first intermediate host (Mas-Coma *et al*., 2006; Toledo *et al*., 2019). After the development of daughter and mother rediae, cercariae emerge and swim in the freshwater to reach aquatic plants, where they encyst as metacercariae. Metacercarial encystment can also occur in snails, tadpoles, frogs and crayfishes. Definitive host becomes infected when encysted metacercariae are swallowed with tainted vegetation or with animal products, such as raw or undercooked crustaceans, molluscs or amphibians (Fried *et al*., 2004; Mas-Coma *et al*., 2005, 2006; Toledo *et al*., 2019).

### Epidemiology

First cases of human gastrodiscoidiasis were reported in India. Thereafter, human cases have been found in Burma, Nepal, Pakistan, Myanmar, Vietnam, the Philippines, Thailand, China, Kazakhstan, Indian immigrants in Guyana, Zambia, Nigeria and the Volga Delta in Russia (Yu and Mott, 1994; Mas-Coma *et al*., 2005; Sah *et al*., 2019; Toledo *et al*., 2019). Although *G. hominis* is not a common parasite of humans, high prevalences have been found in some areas, especially in India. Buckley (1939) reported a prevalence of 41% in children from Assam and a prevalence of 27% was recorded in people from Bareilly (Dutt and Srivastava, 1972).

Most of the cases appear to be related to the consumption of tainted vegetables directly collected from ponds and rivers, which is a common practice in some Asian countries (Sah *et al*., 2019) or even of vegetables bought in local markets. Uzairue *et al*. (2013) analysed a total of 250 samples of 7 different vegetables in markets from Ekpoma (Nigeria) and detected that 0.9% of cabbage samples were contaminated with metacercariae of *G. hominis*. Moreover, infection with metacercariae encysted in animal products, such as raw or undercooked crustaceans, molluscs or amphibians also may occur (Fried *et al*., 2004).

### Clinical symptoms, diagnosis and treatment

Human gastrodiscoidiasis is commonly asymptomatic. In heavy infections, epigastric pain, abdominal discomfort, diarrhoea and headache may occur (Toledo *et al*., 2019). Moreover, other symptoms including lymphadenopathy, hepatosplenomegaly or anaemia have been reported in individual patients (Dada-Adegbola *et al*., 2004; Sah *et al*., 2019). At the local level, inflammation at the site of attachment due to dragging with the acetabulum, surface desquamation, infiltration with eosinophils, lymphocytes and plasma cells appears in the lesions caused by the fixation of the parasites to the mucosa. Hypersecretion of mucus and necrosis of the mucous glands are also observed (Mas-Coma *et al*., 2005, 2006).

Diagnosis of human gastrodiscoidiasis is performed by detection of eggs in feces. The egg is operculated, non-embryonated and measuring about 150 × 70 mm^2^ (Fig. 3B) (Mas-Coma *et al*., 2005, 2006; Esteban *et al*., 2019; Toledo *et al*., 2019). Praziquantel is the drug of choice. However, a single dose of 500 mg mebendazole also may be efficient (Dada-Adegbola *et al*., 2004; Mas-Coma *Et al*., 2005).

## Concluding remarks

Human echinostomiasis and gastrodiscoidiasis are among the most neglected intestinal trematode infections. Both parasite infections have been considered as minor diseases mainly confined to some areas of Asia. However, the real prevalence of these infections is not known, which makes necessary further efforts and systematic helminthological surveys to determine the real impact on human health. This is of particular importance considering that several factors such as growing international markets, new eating habits in developed countries or demographic changes may be expanding their risk of infection and prevalence in areas where these diseases were not known. Thus, further studies are required to elaborate new maps of risk and a more detailed follow-up may be useful to gain a better understanding of the current incidence of these diseases.
